# Technical opportunities and challenges in developing total-body PET scanners for mice and rats

**DOI:** 10.1186/s40658-022-00523-6

**Published:** 2023-01-02

**Authors:** Junwei Du, Terry Jones

**Affiliations:** 1 Department of Biomedical Engineering, University of California at Davis, Davis, CA 95616 USA; 2 Department of Radiology, University of California at Davis, Davis, CA 95616 USA

**Keywords:** Total-body PET, Small animal, Mice, Rat

## Abstract

Positron emission tomography (PET) is the most sensitive in vivo molecular imaging technique available. Small animal PET has been widely used in studying pharmaceutical biodistribution and disease progression over time by imaging a wide range of biological processes. However, it remains true that almost all small animal PET studies using mouse or rat as preclinical models are either limited by the spatial resolution or the sensitivity (especially for dynamic studies), or both, reducing the quantitative accuracy and quantitative precision of the results. Total-body small animal PET scanners, which have axial lengths longer than the nose-to-anus length of the mouse/rat and can provide high sensitivity across the entire body of mouse/rat, can realize new opportunities for small animal PET. This article aims to discuss the technical opportunities and challenges in developing total-body small animal PET scanners for mice and rats.

## Introduction

Because of the pathophysiological similarities between vertebrate mammals and humans, vertebrate mammals are widely used as models to study human disease and functions [1–7]. Mouse and rat comprise approximately 99.3% of mammals used in pre-clinical research in the USA [8]. Hence, small animal positron emission tomography (PET) scanners, which have small diameters to obtain high spatial resolution to image the small amount of radiation tracer distributed within small structures of the animal body, were designed in the 1990s, starting with the microPET [9]. Now, some hundreds of small animal positron emission tomography (PET) scanners are installed worldwide. Most major academic medical research centers and pharmaceutical companies have access to and routinely use this technology. Key characteristics of small-animal PET are spatial resolution and sensitivity, which strongly affect the quantitative accuracy and precision of PET imaging. However, it remains true that almost all small animal PET studies using mouse or rat as models are either limited by the spatial resolution or the sensitivity (especially for dynamic studies), or both, reducing the quantitative accuracy and quantitative precision of the results. Furthermore, few scanners have been designed to cover the whole body of the mouse or rat with equal sensitivity [10–13]. Hence, few offer the opportunity to undertake interactive “systems” studies of the whole body using simultaneous kinetic time course for the whole tissues of the body. Such pre-clinical studies are destined to support the development of human total-body PET scanner-based applications by developing and characterizing suitable paradigms [14–17].

Quantitative accuracy is strongly linked with spatial resolution through the partial volume effect, the single largest quantitative error in almost all small animal PET studies [18]. Improving spatial resolution reduces the partial volume effect, thus improving accuracy. Quantitative precision is strongly linked with the number of detected events through the statistical uncertainties that are governed by Poisson counting statistics. Increasing the scanner sensitivity increases the number of detected events, thus increasing the precision of PET measurements. A further benefit of higher sensitivity is that it permits faster dynamic imaging, i.e., increasing temporal resolution [14, 19, 20], which can benefit quantification through improved temporal data to feed into tracer kinetic models, such as in measuring the image-derived input function (IDIF) from a major arterial vessel, i.e., aorta.

The sensitivity of PET scanners can be dramatically increased by increasing the geometric coverage to cover the entire body of the subject [12, 21], using thick crystals with high stopping power, and reducing the gap between detector elements [22–24, 24]. To obtain high uniform resolution across the field-of-view (FOV), high spatial resolution detectors with the ability to record depth-of-interaction (DOI) information are needed [11, 12, 25]. In this article, we will discuss the technical opportunities and challenges in developing optimal total-body small animal PET scanners for mice and rats.

## Status of current total-body small-animal PET

**Fig. 1:**
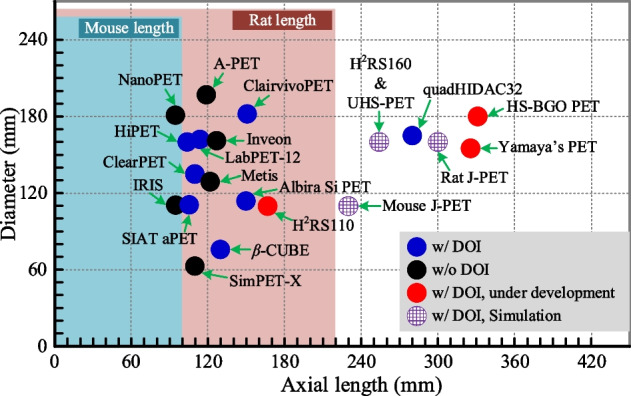
Axial length and diameter of small animal scanners with axial lengths longer than 90 mm [13, 21, 28–31, 38, 40–48]. Only scanners with published results were selected. References of the scanners are shown in Table 3

**Fig. 2:**
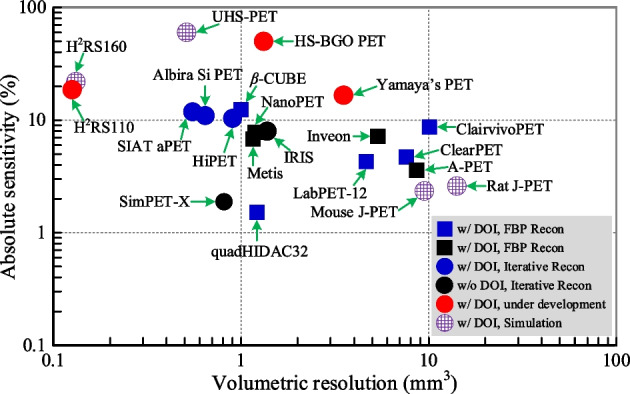
Volumetric resolution and sensitivity at the center of small animal scanners with axial lengths longer than 90 mm [13, 21, 28–31, 38, 40–48], which corresponds to Fig. 1. Only scanners with published results were selected. References of the scanners are shown in Table 3

The axial lengths and diameters of currently available small animal PET scanners with axial lengths longer than 90 mm, together with these under construction and proposed with simulation results, are shown in Fig. 1, while their resolution and sensitivity are shown in Fig. 2. (Only scanners with published results were selected. References of the scanners are shown in Table 3.). Almost all state-of-the-art scanners have an axial length longer than the nose-to-anus length of the mouse, which can be up to 10 cm [26]. However, all the currently available scanners, except the quadHIDAC32, have axial lengths shorter than the nose-to-anus length of the rat, which can be up to 20 cm [27].

The four latest small animal scanners, the β-CUBE, the Albira Si PET, the HiPET, and the SIAT aPET scanners, are all built with DOI detectors and have ~ 10% sensitivities, as measured for a point source at the center of the scanner, and better than the 1 mm resolution. To obtain high spatial resolution across the FOV, all these four scanners are based on detectors with DOI information to reduce the parallax error. The β-CUBE PET scanner incorporates a detector design that comprises a 25.4 × 25.4 × 8 mm^3^ thick monolithic lutetium–yttrium oxyorthosilicate (LYSO) scintillator block coupled to an array of 3.0 × 3.0 mm^2^ Hamamatsu silicon photomultipliers (SiPMs) [28]. A maximum likelihood (ML) clustering algorithm was used to estimate the 3D location of each gamma interaction within the crystals of PET detectors. The detectors have a 1.6 mm DOI resolution. This scanner, with a 76 mm ring diameter and 130 mm axial length, has a 1.1 mm spatial resolution (3D filtered back projection (FBP) reconstruction algorithm) and 12.4% sensitivity (255–765 keV energy window (EW)) at the center of the scanner. The Albira Si PET scanner uses 50 × 50 × 10 mm^3^ monolithic LYSO scintillator blocks coupled to SiPM arrays of 3 × 3 mm^2^ SiPM elements [29]. The scanner has a 114 mm ring diameter, and a 150 mm axial length, and the detectors have around 1.6 mm DOI resolution. The spatial resolution and sensitivity of the scanner are ~ 1 mm (maximum likelihood expectation maximization (MLEM) reconstruction algorithm) and 11.0% at the center of the scanner, respectively. The HiPET scanner is built with dual-layer scintillator arrays based on LYSO and BGO to provide two-layer DOI information [30]. Each scintillator array consists of a front layer, a 48 × 48 LYSO array of 1.01 × 1.01 × 6.1 mm^3^ crystal elements (1.09 mm pitch size), and a back layer, a 32 × 32 bismuth germanate (BGO) array of 1.55 × 1.55 × 8.9 mm^3^ elements (1.63 mm pitch size). The back layer is coupled to Hamamatsu H12700 flat panel multi-anode photomultiplier tube (MAPMT). The HiPET has a 160 mm ring diameter and 104 mm axial length. The spatial resolution is 0.93 mm (3D ordered subset expectation maximization (OSEM) reconstruction algorithm), and the sensitivity is 10.4% (350–650 keV EW) at the center of the scanner. The SIAT aPET is built using dual-ended readout detectors consisting of Hamamatsu S13361-3050-08 SiPM arrays coupled to both ends of LYSO arrays of 1.0 × 1.0 × 20 mm^3^ crystal elements (1.07 mm pitch). This scanner has a 116 mm ring diameter and 105.6 mm axial length. The DOI resolutions of the detectors are around 1.96 mm. The spatial resolution and sensitivity of the scanner are 0.8 mm (3D OSEM reconstruction algorithm) and 11.9% (350–750 keV EW), respectively, at the center of the scanner.

**Fig. 3:**
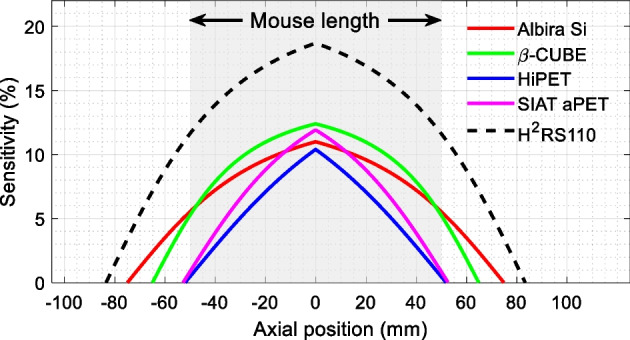
Sensitivity of small animal scanners that can perform mouse studies and have axial FOVs longer than 100 mm. The sensitivities were estimated using the solid angle coverage, and the sensitivities at the center of the scanners are reported using the published data. Note the need to extend the axial length of the scanner beyond the length of the mouse to ensure sufficient sensitivity as realized at the two ends of the animal

However, as shown in Fig. 3, although the HiPET and SIAT aPET scanners have axial lengths slightly longer than the nose-to-anus length of the mouse, they cannot perform total-body studies due to the significant fall-off of sensitivities at the two ends of the scanners [30, 31]. Two-bed positions are needed to image the mouse, and three-bed positions are needed for a rat scan. The β-CUBE and the Albira Si PET scanners can provide higher than 5% sensitivity for the nose and anus with a mouse located at the center of the scanner. Although a sensitivity compromise, they do offer the means to undertake total-body mouse studies.

Among the mice and rats used in the USA, 97.3% of them are mice and 2.7% are rats [8]. Hence, most small animal PET scanners are designed for undertaking mouse studies. However, rats are preferred for studies of the brain due to the larger size of rats and the resolution limitation of currently available PET scanners (Fig. 2) [12, 32–36]. Until recently, the only scanner built to date that can cover the entire rat body is the quadHIDAC32 PET scanner, which has a 280 mm axial length and 165 mm ring diameter [37–39]. The quadHIDC32 PET scanner that was built around 2005 using multiwire proportional chambers (MWPCs) and is still operational has four detector banks arranged in a box shape. Each detector bank has eight separate chambers stacked together to improve the sensitivity and provide a 2.5 mm DOI information, which maintains the spatial resolution across the FOV of the scanner [39]. It has a ~ 1.0 mm resolution (FBP reconstruction algorithm). However, the sensitivity is only 1.5% for a point source located at the center of the scanner, which is much lower than most of the other scanners. The low sensitivity limits the application of the scanner to static PET studies only [38].

## Total-body small-animal PET for mouse and rat

**Fig. 4:**
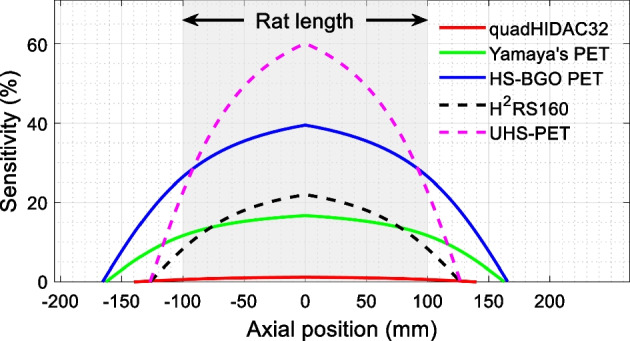
Sensitivity of small animal scanners that can perform both total-body rat and total-body mouse studies. The sensitivities were estimated using the solid angle coverage, and the sensitivities at the center of the scanners were reported using the published data. Note the need to extend the axial length of the scanner beyond the length of the rat to ensure sufficient sensitivity as realized at the two ends of the animal

**Fig. 5:**
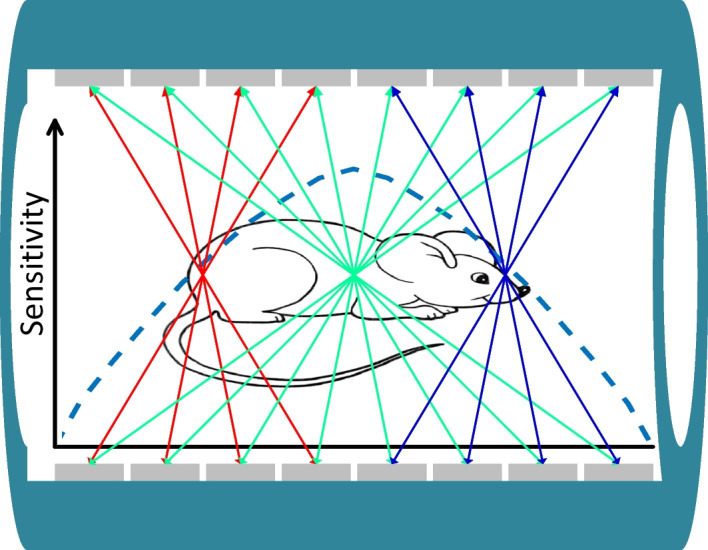
Total-body small animal PET scanners need to have an axial length longer than the length of the mice/rats to maximize sensitivity across the entire body of the animals. The PET scanner (H^2^RS110) shown in this figure, which is designed for total-body mouse studies only, has a 110 mm ring diameter and 167 mm axial length [12]

The function of the PET scanner is to detect the coincident “back-to-back” 511-keV photons that are produced when a positron interacts with an electron [18]. Due to the radiation being emitted isotopically, state-of-the-art PET scanners are all operated in the three-dimensional data acquisition mode to achieve high sensitivity. In three-dimensional mode, the axial sensitivity profile is determined geometrically, which peaks at the center of the scanner and reduces to zero at the two ends of the scanner (Figs. 3, 4, 5) [12, 18]. Hence, to maximize sensitivity for the entire body of the animal, the axial length of the scanner needs to be extended beyond the length of the animals, such as the mouse and the rat, to image the animals using a one bed position for total-body studies (Fig. 5).

## Current developments of total-body small-animal PET

To further increase the performance, especially sensitivity for mouse/rat studies, to our knowledge, up to the moment this review is prepared, six total-body small animal PET scanners are either under development or proposed with simulation results.

Groups from the University of California at Davis and the University of Texas Arlington have proposed a design to build a scanner with a 110 mm ring diameter and 167 mm axial length for total-body mouse studies (Fig. 5) [12]. This scanner is based on dual-ended readout detectors based on position-sensitive SiPMs (PS-SiPMs) coupled to both ends of 20 mm thick LYSO arrays (H^2^RS110 shown in Figs. 1, 2, 3) [12, 51, 52]. Besides increasing the sensitivity, LYSO arrays of 0.44 × 0.44 × 20 mm^3^ crystal elements (0.5 mm pitch size) are used to achieve the physical limits of spatial resolution that could be obtained from coincidence detection. The DOI resolution of the detectors is ~ 2 mm [53]. Monte Carlo simulation results show that this scanner can provide ~ 18.7% sensitivity (250–750 keV EW) at the center of the scanner and better than 10% sensitivity across the entire mouse body. The resolution is ~ 0.5 mm across the whole mouse body, obtained using the MLEM reconstruction algorithm [12].

In Dr. Yamaya’s Lab in Japan, a scanner with a 153 mm ring diameter and 325.6 mm axial length is under construction using four-layer Zr-doped gadolinium oxyorthosilicate (GSOZ) crystal arrays of 2.8 × 2.8 × 7.5 mm^3^ crystal elements [21]. The pitch size of the GSOZ arrays is 2.85 mm, and Hamamatsu H8500 position-sensitive PMTs are used as photodetectors. Depth of interaction is determined using a light-sharing method in which the optical photon distributions arriving at the PMT are modified via the insertion of radial reflectors in the crystal array with different patterns for each layer, providing a 7.5 mm DOI information [54, 55]. The measured sensitivity is 16.7% at the center of the scanner using a 400–600 keV energy window. The spatial resolutions at the center and 30 mm radial offset are 1.52 mm and 1.75 mm, respectively, obtained using OSEM reconstruction algorithm. Due to GSOZ arrays with a large pitch size are used, the spatial resolution of this scanner is worse than most of the state-of-the-art scanners (Fig. 2), such as the β-CUBE and the Albira Si PET scanners.

At the University of California at Davis, we are also building a similar axial length but wider bore scanner with a diameter of 180 mm and axial length of 331.2 mm (HS-BGO PET in Figs. 1, 2, 4) [49]. Dual-ended readout detectors are used based on 32 × 32 BGO arrays with a 1.6 mm pitch size and 20 mm thickness [56]. BGO scintillator, instead of widely used L(Y)SO, is selected for very-low-dose PET imaging. Compared to L(Y)SO, BGO has much lower intrinsic radiation [57], higher stopping power, and higher photoelectronic ratio [18, 58, 59]. These make BGO a better choice for high sensitivity small animal PET scanners, especially for very-low-dose studies, such as cell tracking using long lived radionuclides [60, 61] and gene expression [62–64]. The lower cost of BGO will also make the scanner more affordable. The measured results show that the detector has a ~ 3.2 mm DOI resolution [56]. From the Monte-Carlo simulation, the sensitivity of the scanner is 39.5% at the center of the scanner (250–625 keV EW), and higher than 20% within the 200 mm axial center region for rat studies, and higher than 30% within the 100 mm axial center region for mouse studies (Fig. 4). The spatial resolution is ~ 1.1 mm across the entire mouse/rat body. However, due to the slower decay time of BGO and the larger size of the detector module, the peak count rate is low, which makes the scanner a good choice principally for low activity studies.

The HS-BGO PET scanner is designed to work optimally for studies with body loads of radioactivity lower than 50 uCi (1.85 MBq). This is due to the large size of the detector (51.2 × 51.2 mm^2^), the slow decay time of the BGO, and the signal multiplexing readout which is chosen to simplify the readout electronics [65]. To build PET scanners that can be used both for total-body mouse and rat studies, and have high resolution and high sensitivity simultaneously, we also proposed to build two high performance PET scanners (H^2^RS160 and UHS-PET scanners shown in Figs. 1, 2, 4) that can work with high injected dose. They both have a diameter of 160 mm and axial length of 254 mm and are based on dual-ended readout detectors to obtain DOI information. The H^2^RS160 PET scanner is based on linearly graded SiPM (LG-SiPM, a type of PS-SiPM) arrays coupled to both ends of LYSO arrays with a 0.5 mm pitch size and 20 mm thickness [12, 52]. The DOI resolution of the detectors is ~ 2.0 mm [31]. The Monte Carlo simulation results show that the H^2^RS160 PET has 22% sensitivity (250–750 keV EW) at the center of the scanner. The sensitivity across the mouse body is higher than 16%, and it is higher than 7% across the entire rat body. The spatial resolution obtained using the MLEM reconstruction algorithm is ~ 0.5 mm across the mouse/rat bodies. The UHS-PET scanner is based on our recently developed curved LYSO arrays with a 1.0 mm pitch size on the inner side and 30 mm thickness [22, 66]. The curved LYSO arrays were developed to reduce the dead space between detector modules in PET scanners with conventional cuboid scintillator arrays, and thereby the sensitivity can be significantly improved (Fig. 7). The dual-ended readout method was also used to obtain a ~ 2.8 mm DOI resolution from 30 mm thick LYSO [22]. Based on the measured detector results, Monte Carlo simulation shows that the scanner can provide a ~ 0.8 mm spatial resolution across the mouse/rat body using the MLEM reconstruction algorithm. The sensitivity is 60.1% (250–750 keV EW) at the center of the scanner, and it is higher than 45% across the mouse body and higher than 20% across the rat body [50].

To build cost-effective total-PET scanners, researchers at the Jagiellonian University introduced plastic scintillator based PET scanners (J-PET) [67, 68]. Opposed to the traditional PET design concept, J-PET uses axially arranged detection units, each consisting of a plastic scintillator strip readout with silicon photomultipliers on both ends. Although the J-PET technology was initially developed for clinical PET scanners, recently, the sensitivity of a total-body mouse J-PET and a total-body rat J-PET were simulated using the GATE software [48]. The mouse J-PET scanner is based on 1 × 1 × 230 mm^3^ EJ-230 plastic scintillators, and has a ring diameter of 110 mm and an axial length of 230 mm. The rat J-PET scanner is based on 1 × 1 × 300 mm^3^ EJ-230 plastic scintillators, and has a ring diameter of 160 mm and an axial length of 300 mm. The volumetric spatial resolutions of the mouse J-PET and rat J-PET are 9.5 mm^3^ and 14.1 mm^3^, respectively. The sensitivities at the center of the FOV of the mouse J-PET and rat J-PET are 2.35% and 2.6%, respectively. These spatial resolutions and sensitivities are lower than those of the scanners described above. The low sensitivities are mainly caused by the low intrinsic stopping power of the EJ-230 plastic scintillator. Although the mouse J-PET and rat J-PET have large FOVs to cover the entire body of mice and rats, the low sensitivity could be a limitation for dynamic studies and for imaging low level of tracer. To improve the sensitivity, one possibility would be to stack more detector modules in the radial direction.

## Opportunities for total-body small-animal PET

### The limitations of scanners with short axial FOV

Small animal PET scanners with short axial lengths have lower cost, which are cost effective tools to obtain dynamic or static images of pre-selected regions of the animals [69, 70], or to obtain total-body static images of the entire mouse/rat using multi-bed approaches [31]. However, the drawbacks of the scanners with short axial FOV are also obvious: (1) they cannot, or at least cannot easily perform total-body dynamic imaging and hence the creation of total-body functional parametric images [71], (2) they minimize the opportunity for studying in real time across body interactions, and (3) their sensitivity is usually low due to the small solid angle coverage [10–12].

#### Total-body image

Total-body imaging using one bed position is required for applications in which the temporal distribution of radiotracer in the entire body or multiple organ systems is of interest. It provides the means to obtain the arterial image derived input function (IDIF) from the heart or aorta, which will be within the FOV of the scanner all the time. This cannot be done, or at least cannot be easily done, using multi-bed dynamic imaging in small animal studies and will certainly compromise the ability to obtain high image quality [72, 73]. Another example is the opportunities for systems biology research [16, 74, 75] which, although still in its infancy, has the prospects for undertaking new areas of biological research. However, to explore the broad scope for such investigations, there is clearly a need to first characterize/validate such pilots at the pre-clinical level. In the systems biology approach, multi-organs need to be imaged simultaneously to study the interactions between different organs such as the brain-gut, and brain–heart. It is envisaged that pre-clinical small animal PET-based developments, including the formulation of investigative paradigms and protocols, could be undertaken to develop and validate new kinetic models for quantifying these interactions.

#### Low sensitivity

The signal-to-noise ratio (SNR) of the image is determined by the number of detected events. Low sensitivity is the main limitation, especially for realizing high-resolution and dynamic imaging studies with tracer kinetic modeling, because short-time-frame datasets are always statistically limited. To obtain sufficient events, injection doses of high levels of radiolabeled tracer and/or long acquisition time are requested [69, 76], especially for multi-bed static imaging or dynamic imaging. However, to compensate for this, high levels of a radiolabeled tracer especially those produced with low specific activity, namely where there are associated large levels of cold compound, cannot be used. As is could no longer be considered, a procedure adheres to the tracer principle, in which the physical amount of tracer does not perturb the biological process being traced, and could lead to physiologic effects and nonlinear kinetics [77, 78]. The key purpose of developing small animal PET scanners is to study the disease models that can be translated to human later. To have the similar physiologic effects, many studies suggest that the radiopharmaceutical injection dose be scaled either by the body weight or by the body surface area [79]. In human studies, the typical injected fluorodeoxyglucose (FDG) dose is around 10 mCi. If assuming the patient is 175 cm tall, to have a similar physiologic effect, the injected does scaled down by body weight for a 10 cm long mouse should be around 1/5000 of that for human, which is ~ 2 µCi, far less than the ~ 200 µCi injected doses widely used for mouse studies nowadays. However, if a small animal (mouse or rat) and clinical PET study are to be “equivalent” from an imaging point of view, the same coincidence rate must be observed from a small animal image voxel as from a clinical image voxel [77]. The spatial resolution of state-of-the-art clinical PET scanners is ~ 3 mm; hence, for small animal PET scanners with ~ 1-mm spatial resolution, the sensitivity of the small animal PET scanners should be 27 times of those of the clinical PET scanners. Most of the clinical PET scanners with < 30 cm axial FOV have ~ 1 to 2% sensitivity [80–83]. Hence, small animal PET scanners should ideally have a sensitivity better than 50%, which is far beyond the ~ 10% sensitivity of currently available small animal PET scanners (Fig. 2). One effective way to increase the sensitivity and reduce the injected dose is to increase the solid angle coverage by increasing the axial FOV of the scanner [19].

### The benefits of total-body small animal PET to applications

All studies based on mice and rats as models can benefit from the high sensitivity and large geometric coverage of total-body small animal PET scanners. With the improved sensitivity, we can obtain better image quality, or use shorter scan time or less radiation dose, or image the animal over extended time periods [19, 20, 84–86]. Examples are fast dynamic imaging to study the time courses of a label in tissue regions of interest and in arterial blood [20], and studies of chronic inflammatory disease where the binding of translocator protein (TSPO) ligands can be low and hard to detect [87–89]. Another key stimulus for realizing increased sensitivity is the limitation as to the amount of tracer that can be administered of molecules that are with selectivity to specific cellular binding sites. Since it is important to maintain the tracer principle, it is clearly necessary to ensure the amount of the carrier molecules administered along with the radiolabeled molecule does not perturb those binding sites. In effect, there will often be limits as to how much tracer and its accompanying carrier molecule can be administered [90]. Hence, the need for high detection sensitivity to ensure proficient studies can be undertaken in the laboratory mouse or rat in the presence of the described restrictions.

Meanwhile, studies using the recently developed human total-body EXPLORER PET scanner have shown the benefits of total-body PET scanning using one-bed position [20, 86, 91], which are pushing human PET scanning to a new level. Several EXPLORER scanners have been installed, and more long axial FOV human scanners are under development [15, 92, 93]. Total-body PET scanners are destined to affect a step change in the application of PET based molecular imaging in clinical research and healthcare. They represent the only radiological technology that is able to simultaneously image the whole of the human body. Based upon the tracer principal to measure regional tissue function, the means is offered for undertaking a “systems” study of the human by relating for example how specific tissues functionally interact in health, disease and in response to therapy. It is foreseen that such system research studies are destined to impact key areas such as the immune therapy of cancer, viral immunity, drug development through pharmacokinetics and pharmacodynamics studies, brain-body interactions, auto immune conditions, and cardiovascular disease.

Several high-resolution and high-sensitivity human brain PET scanners are also under development for improving human brain imaging [94–97]. Both human total-body PET, the applications of which are still in their infancy, and more established brain PET need a homologous pre-clinical tool using mouse or rat models to develop, validate, and characterize the paradigms and protocols that can be translated into human PET studies, which are currently unavailable.

Total-body small animal PET scanners can provide high sensitivity across the entire body of mouse/rat with high spatial resolution, thereby providing new opportunities for small animal PET research, and/or opening new windows to using PET for currently unknown applications. Overall, advanced scientific tools offer higher opportunities for exploratory studies.

#### Image-derived input function

One emerging example of the application of high-resolution and high-sensitivity total-body small animal PET is to obtain the noninvasive IDIF for kinetic modeling. Kinetic modeling with dynamic PET provides sophisticated quantitation of the tracer kinetics with improved prognostic and predictive values over those of simple standard uptake value (SUV) measurements [98–100]. The arterial input function, which describes the time-activity curve of the radiotracer in the blood, is a prerequisite for kinetic modeling. The input function can be derived using arterial blood sampling, which is the conventional method [101]. However, because of the limited blood volume of mouse and rat, the related difficulties in withdrawing blood, and the effects of the accrual rates of the animals, IDIF is an elegant and alternative way, which can be derived from the dynamic images of the arterial blood pools included in the field-of-view of PET [98, 99]. For mouse or rat based studies, the most reliable blood pools for IDIF measurements are the left ventricle and atrium, and ascending or descending aorta, which are the larger blood pools but inside mouse or rat, the sizes are very small [102]. To image these blood pools with a high efficiency during the dynamic studies, the PET scanners need to have high sensitivity which can be derived from an axial field-of-view that is longer than the length of the animals. To reduce the partial volume effect (PVE), spill in and spill out from surrounding tissues of the blood pool and thereby increase the quantitation accuracy and precision of the IDIF [103], both high spatial resolution and high sensitivity are required. This is especially the case for the early time points of a kinetic study, where there are fast changing concentrations of tracer within the blood pool and at the later time points where the levels are low.

### Advancing the technology of small animal imaging

The performance of the PET scanner is mainly dominated by the detectors and the readout electronics. During the past 2 decades, both the detector technologies and readout electronics have changed rapidly, which opens the door for high-performance small animal PET scanners.

#### PET detector

Different types of detectors have been developed [18], including scintillator- and photodetector-based detectors [21, 104–106], semiconductor-based detectors [107–109], and gas detectors [38, 110, 111]. Semiconductor-based detectors have good energy resolution; however, their timing resolutions are poor, in tens of nano-second [108, 112]. Cerenkov photondetection has been proposed to improve the timing resolution. However, because of the limited numbers of emitted Cerenkov photons which typically for BGO is around 15, it is still in the early stage, and not ready for use at the scanner level, or even at the detector level [113, 114]. The major problem of gas detectors, such as the multigap resistive plate chamber (MRPC) and MWPC, is their low detection efficiency, even when several detectors are stacked together [38]. Its timing resolution and energy resolution also cannot compete with scintillator- and photodetector-based detectors [115–117]. Hence, most of the state-of-the-art PET scanners are based on scintillator- and photodetector-based detectors [19, 28, 30, 31, 93, 118, 119].

#### Scintillator

The emergence of LSO in 1992 revolutionized the PET scanner in various aspects [11, 120]. The first small animal PET, the microPET developed in the middle of 1990s, was the first to take advantage of the LSO scintillator. Among all the scintillators used in PET today, LSO and LYSO (a variation of LSO), which have similar characteristics, are still the best choice for most applications. This is due to their high density, high light yield, high stopping power, and fast decay time (Table 1), which can provide the overall best trade-off between spatial resolution, timing resolution, and energy resolution. However, due to the intrinsic radiation caused by ^176^Lu [57, 121, 122], scanners based on LSO and LYSO are not an ideal choice for ultra-low activity studies. BGO, due to its much lower intrinsic radiation and slightly higher stopping power and photoelectric fraction (Table 2), is a better choice for scanners aiming for low activity imaging.

**Table 1:** Peak noise equivalent count rates (NECRs), system type, and references of scanners shown in Figs. 1 and 2

Name of scanner	Peak NECR (kcps)		System type	References
	Mouse phantom	Rat phantom		
NanoPET	430	130	PET/CT	[45]
A-PET	~ 500	~ 230	Standalone	[40]
Inveon	1670	590	PET/CT	[43]
Metis	1344	640	PET/CT	[47]
IRIS	185	40	PET/CT	[46]
ClairvivoPET	415 kcps (50 mm ^18^F line source)		Standalone	[41]
HiPET	179	63	Standalone	[30]
LabPET-12	362	156	PET/CT	[42]
ClearPET	Unknown	Unknown	PET/CT	[44]
Albira Si PET	486	239	PET/SPECT/CT	[29]
SIAT aPET	324	144	MR-Compatible	[31]
β-CUBE	300	160	PET/CT	[28]
SimPET-X	348	Unknown	MR-Compatible	[13]
quadHIDAC32	67	52	Standalone	[38]
Yamaya’s PET	Unknown	Unknown	Standalone	[21]
Mouse J-PET	Unknown	Unknown	Unknown	[48]
Rat J-PET	Unknown	Unknown	Unknown	[48]
HS-BGO PET	Unknown	Unknown	MR-Compatible	[49]
H^2^RS110	Unknown	Unknown	MR-Compatible	[12]
H^2^RS160	Unknown	Unknown	Unknown	[12]
UHS-PET	Unknown	Unknown	Unknown	[50]

**Table 2:** Properties of part scintillators used in PET [18, 123]

Properties	BGO	L(Y)SO	GSO	GAGG^a^
Density (g/cc)	7.1	7.4	6.7	6.6
Effective atomic number	73	66	59	–
Decay time (ns)	300	40	60	50–150
Light yield (photons/MeV)	8000	20,000–30,000	12,000–15,000	30,000–50,000
Attenuation length at 511 keV (mm)	10.4	11.4	14.1	–
Photoelectric fraction (%)	40	32	25	–
Index of refraction	2.15	1.82	1.85	1.91
Peak emission (nm)	480	420	430	520–530
Intrinsic radiation	No	Yes	No	No

^a^Date from https://www.epic-crystal.com/oxide-scintillators/gagg-ce-scintillator.html. GAGG with different components shows different performances

**Fig. 6:**
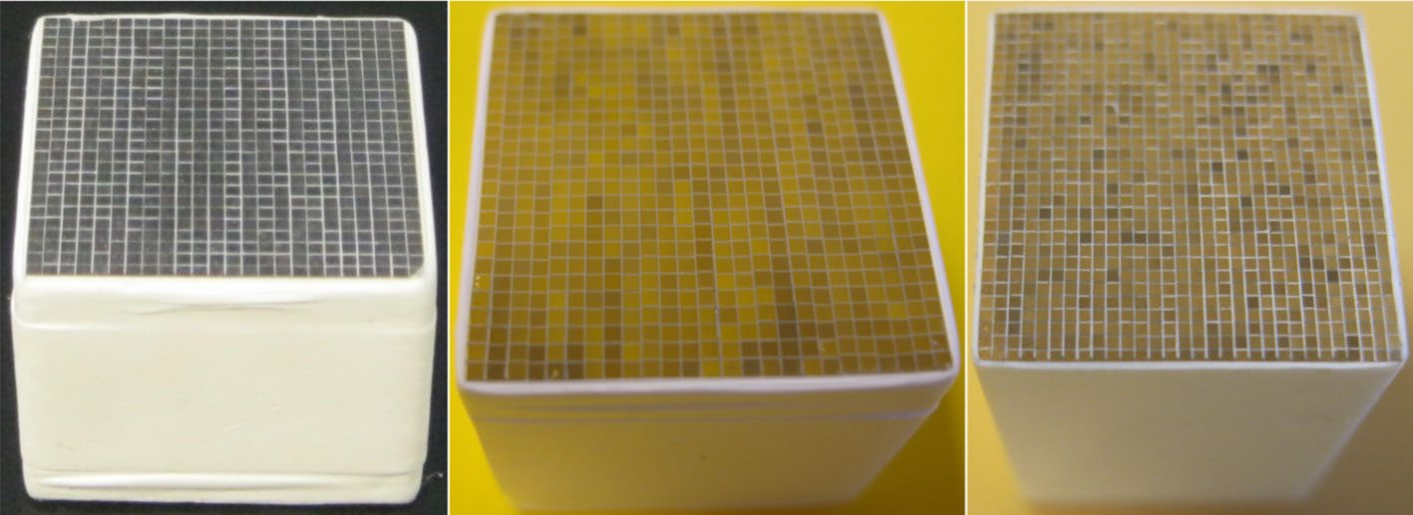
LYSO arrays with different pitch sizes. (left) 1.0 mm pitch size, (middle) 0.75 mm pitch size, and (right) 0.5 mm pitch size [52, 128, 129]

**Fig. 7:**
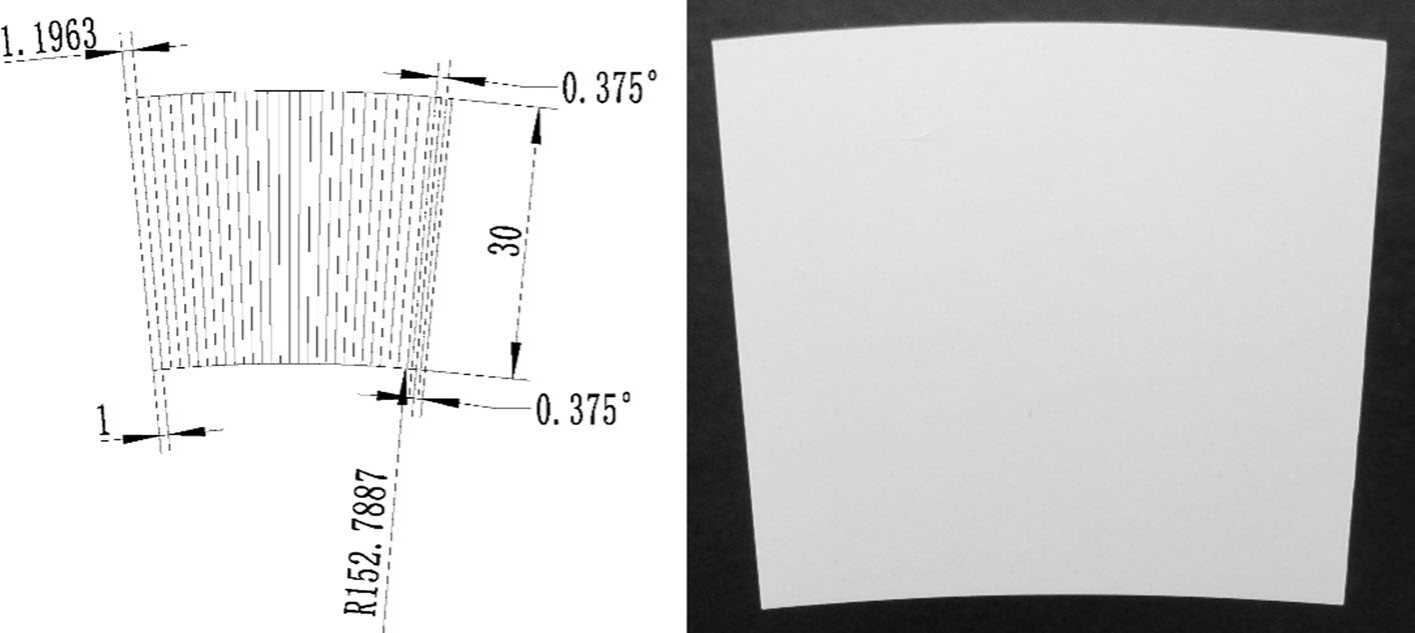
(Left) Schematic and (right) photograph of a curved shape LYSO array used to minimize gaps between detector blocks [22]

*Scintillator array* Scintillator arrays used in high-resolution small animal PET scanners always have a pitch size smaller than ~ 1 mm [69, 70, 119]. Much effort has been spent by different researchers to optimize the scintillator arrays, such as the surface treatment [56, 124, 125], the reflector [53, 124, 125], and the array fabrication method [126, 127]. At the University of California at Davis, during the past 20 years, working with different companies, we have optimized the fabrication method and fabricated different scintillator arrays with pitch sizes down to 0.25 mm (Fig. 6) [52, 128, 129] using different materials [52, 56, 130] and with different shapes (Fig. 7) [22, 130, 131]. Now, we can fabricate high-quality scintillator arrays with small pitch sizes for different applications.

*Monolithic scintillator* Monolithic scintillator based detector is another attractive approach. Compared to scintillator array-based detectors, the major advantages of monolithic scintillator-based detectors are that the monolithic scintillator is easier to fabricate and the elimination of dead space due to reflector between detector elements—lending to potentially high sensitivity. The drawbacks are that more complicated position algorithms, such as deep learning or maximum likelihood method, are needed, and corrections for edge effects in the block are challenging [132, 133]. Different groups have been working on the optimization of the monolithic scintillator based detectors [132–136] and are showing improved performance. Several pre-clinical PET scanners based on monolithic detectors have been developed [28, 29, 134], which provides a way to scale up these scanners to total-body mouse/rat scanners. However, a tradeoff between spatial resolution and sensitivity is required and challenging to make. To obtain high sensitivity, 20–30 mm thick crystals are preferred. However, to obtain high spatial resolution with the monolithic scintillator, thin crystals are required. The β-CUBE PET scanner uses 8 mm thick LYSO, and the Albira Si PET scanner uses 10 mm-thick LYSO to obtain ~ 1 mm spatial resolution and ~ 10% sensitivity [28, 29]. It would be difficult to improve the positioning accuracy and hence spatial resolution without sacrificing the sensitivity, and vice versa.

#### Photodetector

**Fig. 8:**
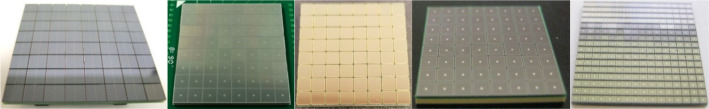
SiPM arrays with different pixel sizes. (from left to right) 8 × 8 array of 6 × 6 mm^2^ SiPMs from SensL, 8 × 8 array of 4 × 4 mm^2^ SiPMs from Hamamatsu, 8 × 8 array of 3 × 3 mm^2^ SiPMs from KETEK, 8 × 8 array of 2 × 2 mm^2^ SiPMs from Hamamatsu, and 16 × 16 array of 1 × 1 mm^2^ SiPMs from Hamamatsu [56, 128, 129, 139]

Earlier PET scanners used PMTs as photodetectors; however, most state-of-the-art PET scanners currently use SiPMs as the photodetector [19, 31, 127, 137]. SiPMs and PMTs have similar gains of around 10^6^. Compared to PMT, the advantages of SiPMs are their low working bias voltage (lower than 100 V), low noise, good single-photon timing resolution, compact size, and insensitive to magnetic fields [137]. Each SiPM pixel, consisting of hundreds to tens of thousands of microcells with sizes ranging from 5 to 100 um [138], can be fabricated in arrays of different sizes (Fig. 8) [56, 104, 128, 129, 139]. These SiPM arrays can be fabricated by arranging multiple SiPM pixels on various printed circuit boards (PCBs).

**Fig. 9:**
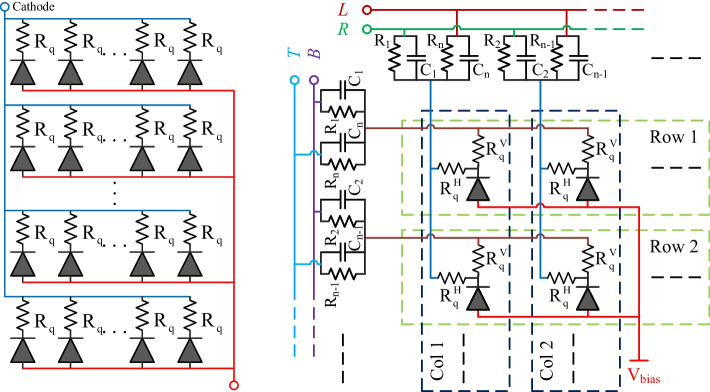
Schematics of (left) non-PS-SiPM and (right) linearly-graded SiPM (LG-SiPM). LG-SiPM is a type of PS-SiPM [52]

SiPMs are semiconductor devices that can be fabricated in most semiconductor foundries. Compared to PMT, the fabrication of SiPMs is much easier, which also leads to a lower cost. At this time, more than ten companies are involved in developing SiPMs. The performance of SiPMs has changed rapidly during the past 10 years resulting in mature products. Today, a range of high-performance SiPMs are provided by different companies (Figs. 8, 9). SiPM has been accepted as the standard photodetector by the PET community and revolutionized PET technology [137].

**Fig. 10:**
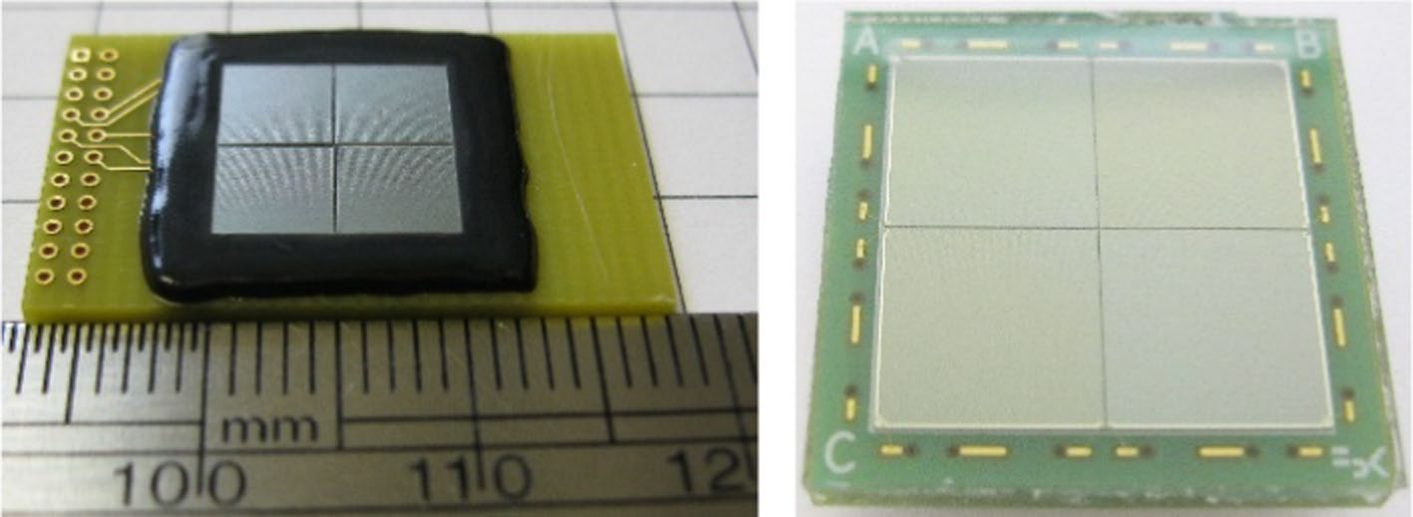
Photographs of 2 × 2 PS-SiPM arrays from (left) Radiation Monitoring Devices, Inc. (RMD) [140, 143] and (right) Fondazione Bruno Kessler (FBK) [52]

To develop SiPM for super-high-resolution PET applications, PS-SiPMs were invented (Figs. 9, 10) [52, 140–142]. For non-PS-SiPM (as these shown in Fig. 8), the anodes and cathodes of all the microcells in one SiPM pixel are connected to form one common anode and one common cathode (Fig. 9). In this way, the fired microcells of the SiPM pixel cannot be located. To encode each microcell, a position encoding circuit for each microcell can be intergraded inside the SiPM. Different types of PS-SiPMs have been developed, and LYSO arrays with a 0.5 mm pitch size and 20 mm thickness have been clearly resolved [52]. Using PS-SiPM, small animal PET scanners with approaching the physically limited spatial resolution can be developed [12].

One critical disadvantage of the SiPM, compared to PMT, is the greater temperature dependency of the SiPM. Both the breakdown voltage and noise of SiPM change with the temperature. The lower the temperature, the lower the noise and the breakdown voltage. However, the animals are typically scanned under anesthesia and require heated beds to preserve physiological functions. To maintain the gain and keep the noise at an acceptable level, cooling, either using water or air, to minimize the temperature variation is necessary.

#### Readout electronics

As SiPMs are accepted as the standard photodetectors by the PET community and are widely used in different PET scanners, a range of application-specific integrated circuits (ASICs) or data acquisition systems (DAQs) have been developed for SiPM based PET scanners by different research groups or companies [144]. Examples for ASICs are the PETsys TOFPET ASIC [145], the HRFlexToT ASIC [146], and the PETA4 ASIC [147]. Companies like PETsys Electronics and Hamamatsu also provide complete data acquisition system solutions based on their ASICs for PET scanners [148, 149].

ASIC-based data acquisition systems have the advantages of low power and compact size; however, they are not flexible. To design flexible readouts for developing early prototype PET scanners, field-programmable gate array (FPGA)-based readouts, such as the FPGA-based Sigma-Delta analog-to-digital converter (ADC) readout system [150, 151] and FPGA-based comparators and time-to-digital converter (TDC) have been developed [152].

All these advances in readout electronics can be used for the development of total-body small animal PET scanners.

## Challenges

### Spatial resolution

**Fig. 11:**
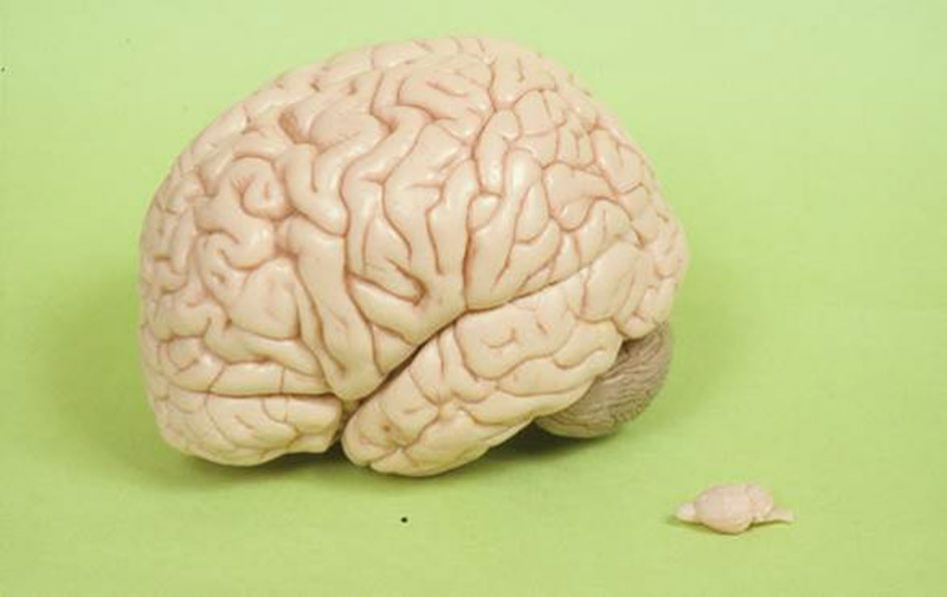
Comparison of a human brain and a rat brain

Spatial resolution determines the finest anatomical structure that the scanner can resolve and still provide quantitative accuracy needed for measuring tissue concentration of its tracer concentration as limited by the partial volume effect. The lengths of most human bodies range from 150 cm to over 180 cm [153], while most of the laboratory used mice and rats have nose-to-anus-lengths smaller than 10 cm and 20 cm, respectively. The human brain has a linear diameter of ~ 11.0 cm, while the linear dimensions for mouse and rat are ~ 12 mm and ~ 8 mm (Fig. 11), respectively. Currently, clinical PET scanners have spatial resolutions as good as ~ 3.0 mm [19, 91], and dedicated human brain PET scanners with ~ 1-mm spatial resolutions are under development [95]. To obtain the same quantitative accuracy in mouse/rat studies as in human studies, PET scanners for total-body mouse and rat studies should have resolution better than 0.2 mm and 0.4 mm, respectively; for mouse/rat brain studies, the scanners should have resolution better than 0.1 mm. However, this is far beyond the spatial resolution of currently available small animal PET scanners (Fig. 2) [10–12].

The fundamental resolution of the PET scanner (*R*_sys_) is determined by the detector resolution (*R*_det_), the positron range (*R*_range_), and the diameter of the scanner (*D*) [18, 154], which can be estimated using formula

1 $$R_{{{\text{sys}}}} = \sqrt {R_{\det }^{2} + R_{{{\text{range}}}}^{2} + (0.0022D)^{2} }$$

where 0.0022*D* is caused by the noncollinearity of annihilation photons. To obtain higher system resolution, PET scanners need to have smaller ring diameters and use higher spatial resolution detectors.

**Fig. 12:**
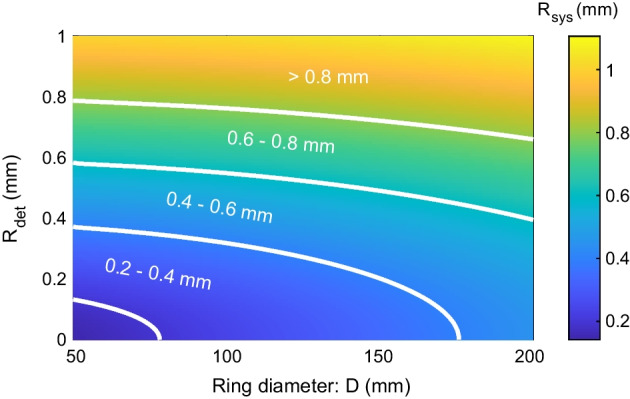
Fundamental spatial resolution versus ring diameter and detector resolution, assuming a 0.102 mm FWHM positron annihilation point distribution from the ^18^F label source [155]. The resolutions are worse for larger ring diameters, which is caused by the noncollinearity of the annihilation photons as shown in formula (1) [18]

For ^18^F, which is the most widely used radionuclide today for labeling PET tracer molecules, the full width at half maximum (FWHM) of the positron annihilation point distribution is 0.102 mm (*R*_range_) [155]. Then, the fundamental resolution of the scanner versus the detector resolution (*R*_det_) and ring diameter (*D*) is shown in Fig. 12. It is obvious that it is impossible to achieve 0.1 mm spatial resolution, unless we will find radionuclides with much smaller positron ranges in the future. The ring diameter can be reduced; however, it needs to be large enough to fit the animals into the scanner. A smaller diameter will also cause a larger parallax error (DOI effect). Hence, most of the PET research focuses on developing high-resolution PET detectors to improve the system-level resolution [52, 70, 107, 134, 156]. The most successful high resolution detectors which have been used at the scanner level were designed using scintillator arrays with ~ 0.5 mm pitch size [69, 70]. However, further reducing the pitch size will reduce the detection efficiency of the detector through reducing the active area of the individual detector elements [12]. The loss of spatial positioning due to the photon scattering within the detector block will be larger for scintillator arrays with smaller pitch sizes, too. For monolithic crystal-based detectors, to obtain higher detector resolution, thinner crystals are required, which reduces the detection efficiency.

### Depth-of-interaction information

**Fig. 13:**
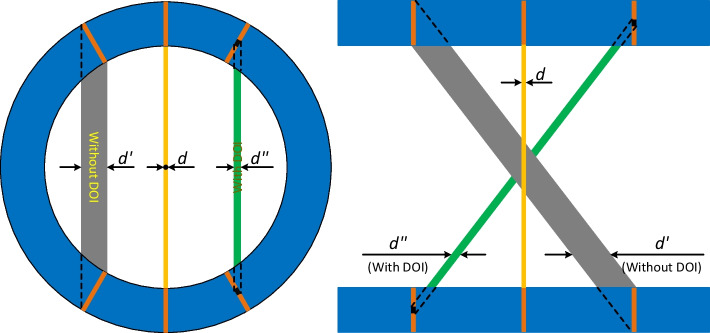
Illustrates how the depth of interaction parallax effect deteriorates spatial resolution. The resolution of the scanner can be improved both in the radial direction and axial direction by obtaining DOI information. *d* is the width of the detector element in the scintillator array

To provide sufficient stopping power for the incoming gamma photon, detectors based on scintillator arrays or semiconductors need enough thickness, which causes the well-known parallax error, both in the radial direction and the axial direction (Fig. 13). The parallax error results in the resolution deteriorating as one moves away from the center of the scanner [25, 70]. To reduce the effect of parallax error, DOI information of the gamma photon interaction positions is needed [25]. In effect, to obtain uniform high resolution across the FOV, the DOI resolution needs to be at least as good as the detector resolution. Different methods have been proposed to obtain the DOI resolution [11, 157]. These include using monolithic scintillator [136, 158], multilayer scintillator [105, 159–163], customized reflector designs [116, 164, 165], and the dual-ended readout method [22, 22, 56, 166]. While current detectors based on scintillator arrays can have a resolution as good as 0.5 mm [52, 53, 156], it is still difficult to obtain the equivalent 0.5 mm DOI resolution without sacrificing other performance such as by reducing the thickness of the scintillator or significantly increasing the complexity of the detector. The best DOI resolution of 0.77 mm has been reported using the X’tal cube by segmenting the scintillator to 0.77 × 0.77 × 0.77 mm^3^ elements and reading out from six sides of the scintillator block [163]. However, the complexity and cost of this detector are high, and the dead space between detector modules and hence sensitivity loss will be large if these detectors were to be used to build scanners.

Good DOI resolution is needed to obtain the best spatial resolution using thick scintillators. DOI resolutions of ~ 2 mm have been reported for 20 mm thick LYSO detectors [65], and ~ 1.2 mm DOI resolutions were obtained from 20 mm thick GAGG scintillators [167]. To date, the best achieved spatial resolution is ~ 0.5 mm using an LYSO array-based detector [52, 69, 70]. Hence, further significant effort is needed to improve the DOI resolution.

### Timing resolution

Time-of-flight (TOF) information can be used to improve the effective sensitivity of PET by a factor of $\frac{L}{{1.6c\delta_{t} }}$, where $\delta_{t}$ is the timing resolution, *L* is the size of the animal, and *c* is the light speed [168–171]. Better timing resolution also can reduce the random coincidence event rate by using a narrower timing window [18], and maybe more interesting for direct positron emission imaging that eliminates the tomographic reconstruction, which introduces statistical noise [172]. However, the best timing resolution currently obtained using 20 mm thick L(Y)SO is ~ 100 ps [173]. For this, a pair of LSO detector elements with a size of 2 × 2 × 20 mm^3^ were used and read out using an oscilloscope with a bandwidth in GHz and a sampling rate of over giga-samples per second (GSPS). The timing resolution will be worse when the crystal cross-section area becomes smaller. This is because as within smaller crystals, the scintillation photons have more reflections, which causes more scintillation photon loss, before reaching the photodetectors, due to the increased reflection numbers on the four lateral sides of the crystal. At the moment, most of the small animal PET scanners still have a timing resolution in several nano-seconds [28, 30, 31], which is not useful for time-of-flight PET. Even 100 ps timing resolution, which corresponds to 3 cm in the distance, is not good enough for small animal PET studies. Improving the timing resolution of high-resolution small animal PET, such as good as 10 ps, to be as equivalent to what has been achieved for human PET scanners in increasing signal to noise is still a challenge and a long-term goal. Even when we have a 10 ps timing resolution, we still need image reconstruction for small animal PET scanners, as 10 ps timing resolution corresponds to 1.5 mm spatial resolution, which is not as good as current small animal PET scanners.

### Inter-detector and inter-crystal scatter

Compton scatter, which causes inter-detector scatter and inter-crystal scatter in PET, is an unfavorable type of photon interaction. The inter-detector scatter happens when one gamma photon interacts with multiple detectors, which results in a loss of sensitivity or multiple coincidences (reducing image quality) [174–176]. The inter-crystal scatter happens when one gamma photon deposits its energy in multiple crystal elements within one detector. This deteriorates the spatial resolution, and hence image quality. [176–178]. The ratios of inter-detector scatter and inter-crystal scatter can be reduced using scintillators with higher density where the higher effective atomic number reduces the Compton scatter probability. However, none of the currently available scintillators has a negligible Compton scatter probability. The Compton scatter probability of L(Y)SO is 68%, and 60% for BGO [123]. The inter-detector scatter ratio also depends on the size of the detector module, where the larger the detector, the lower ratio of the inter-detector scatter. The inter-crystal scatter ratio also depends on the cross-section size of the crystal elements. The smaller the crystal, the higher ratio of the inter-crystal scatter. Hence, for high resolution small animal PET scanners, especially those based on scintillator arrays with small pitch sizes, inter-detector scatter recovery and inter-crystal scatter recovery are both required to improve the sensitivity and resolution, respectively.

Different algorithms have been proposed for inter-detector scatter recovery and inter-crystal scatter recovery [171, 179–181]. However, it is still challenging to do it, especially for high-resolution PET scanners. High-resolution detectors always use light-sharing or charge-sharing methods to improve the spatial resolution and center-of-gravity method to calculate the interaction position [52, 104, 128]. The presence of inter-crystal scatter causes the crystal positioned by the center-of-gravity method is likely to be different from the initial interacted crystal.

Most of the currently available small animal scanners do not implement inter-detector and inter-crystal scatter recovery [28–31]. However, these are needed in order to approach the physically limited spatial resolution and to achieve maximum sensitivity.

### Image reconstruction method

Total-body small animal PET scanners with high spatial resolution have a large number of crystal elements. This is true for both crystal array-based scanners and virtual crystal elements in monolithic crystal-based scanners. For example, the H^2^RS160 PET and UHS-PET scanners consist of 460,800 and 115,200 crystal elements, respectively [12]. If 10-layer DOI information was used, those two scanners would effectively have 4,608,000 and 1,152,000 virtual crystal elements, respectively. Note this is considerably greater than the 2-meter axial long total-body EXPLORER PET scanner with 564,480 crystal elements [19, 182] but with no depth of interaction readout. Hence, highly efficient image reconstruction methods are needed to handle the billions of line-of-responses (LORs). Furthermore, to fully capitalize on the high spatial resolution of a PET scanner, which has optimal geometry for data collection, corrections for the positron range [183, 184] and the inter-crystal scatter should be modeled precisely. In turn, they need to be included in the image reconstructions, which represent ongoing challenges that will require much further effort.

### Scintillator

Although L(Y)SO scintillators have been widely adopted by most of the PET scanners, a major drawback of them is the intrinsic radiation emitted from the ^176^Lu. This radionuclide decays with β^−^ emission into ^176^Hf, together with a cascade of 307 keV, 202 keV, and 88 keV γ photons which are emitted in coincidence [185]. The estimated activity of ^176^Lu in L(Y)SO, is about 240–300 Bq/cm^−3^, which limits the performance of PET scanners based on L(Y)SO for low-dose studies, including cell tracking and gene expression research [11, 60, 61, 64]. As the case for most clinical PET scanners, the lower level of the coincident energy window is set above 350 keV to reduce the effect of the background radiation [122]. However, (1) in small animal PET studies, especially in mouse studies, a threshold lower than 350 keV is preferred to achieve high sensitivity, and (2) even using a tight energy window such as 350–650 keV, the summed energy of the cascade γ photons still can fall into the energy window and degrades the PET image quality. How to optimize the energy window of L(Y)SO-based PET scanners for different applications is a challenging task and needs to be systematically addressed.

Other crystals, such as the recently developed gadolinium aluminum gallium garnet (GAGG) scintillator has a higher light yield than L(Y)SO scintillator [156]. However, GAGG has a lower density, lower stopping power, and slower decay time.

### Electronics

Compared to scanners based on detectors without DOI capabilities, scanners with DOI capabilities require more complex readout electronics to record and process the DOI information. One example is the dual-ended readout detector, which requires 2 times photodetectors. Hence, signal-multiplexing readouts are always used to reduce the electronic channels that need to be digitized [65, 143]. Total-body small animal PET scanners will also produce a large amount of data. The electronics needs to have the ability to acquire and store the data online and reconstruct the images quickly. On the acquisition and storage side, the dead time of the readout electronics needs to be as small as possible, especially when the scanner is used for high count rate dynamic studies to measure the time-activity curves (TACs) from the reconstructed images. On the reconstruction side, total-body small animal PET scanner with DOI information will have a large number of LORs. For a dual-ended readout detector or monolithic crystal-based scanner, the number of possible LORs would be infinite if continuous DOI information is used, which makes it challenging to generate a sensitivity map [186] and increases the required storage and/or memory size. A practical way is to use discrete DOI information based on the discrete DOI resolution. Taken the H^2^RS110 PET as an example, it has 204,800 crystals [12]. The DOI resolution of the detectors is ~ 2 mm. In our simulation, each crystal is divided into 10 virtual crystals with 2 mm thickness in the radial direction. With using this discrete DOI information, the scanner has 2,048,000 virtual crystals with billions of possible LORs, which is still much more than the human total-body EXPLORER PET with 564,480 crystals [19]. Hence, high efficiency image reconstruction methods that can reduce the image reconstruction time and computational load are required, both for crystal array and monolithic crystal-based scanners. However, given the computing power of today’s graphics processing units (GPU) and central processing units (CPU), if economic costs are not a consideration, undertaking the necessary electronic processing is well within the capability of existing technology.

### Mechanical design

One of the goals to design total-body small animal PET scanners is to improve the sensitivity. Hence, detector modules need to be packed tightly to minimize the gap/dead space between detector modules in both axial and transaxial directions to improve the sensitivity. At the same time, the temperature of the detector, especially for temperature-sensitive photodetectors such as SiPM, needs to be controlled.

## Conclusions

The development and construction of small animal PET scanners has established roles in biomedical engineering. The technology has advanced considerably since the reporting of the early systems [9, 187]. However, none of the currently available small animal scanners have both high enough sensitivity to approach the physically limited spatial resolution. However, it is obvious that quantitative accuracy and quantitative precision can be improved by using scanners with identified means for realizing higher spatial resolution and sensitivity. As a result of the efforts from groups in different research fields, it is now possible to increase the performance of small animal PET scanners that promise to open up new application areas of molecular imaging in small laboratory animals. One obvious way to improve the sensitivity to image the whole animal is to build total-body scanners with longer axial lengths than that of the animals. The technologies to enable this are in place, although there are still challenges, such as how to improve the DOI resolution to reduce parallax error and correct for inter-crystal scatter. Dual-ended readout detectors based on PS-SiPM and scintillator arrays are expected to be the most promising candidates for building total-body small animal PET scanners that could approach the theoretically achievable resolution and high sensitivity. We project that the reported under-construction scanner (the HS-BGO PET and H2RS110 PET shown in Figs. 1, 2) with significantly improved sensitivity will open new applications using small animal PET and thereby lead the way to the further development of total-body small animal PET scanners. However, many technical questions and challenges still need to be addressed as have been discussed in this article. However, we suspect many technical opportunities and challenges remain to be uncovered. We look forward to researchers from PET communities, both in the academia and industry, to resolve these challenges to develop the next generations of small animal PET scanners with improved performance, and thereby enhance molecular imaging based research in small laboratory animals.

## Data Availability

Not applicable.

## Appendix

See Table 3.

**Table 3:** Abbreviations of PET scanners shown in Figs. 1, 2, 3, and 4

Abbreviation	Description	References
NanoPET	Commercially available scanner provided by Mediso	[45]
A-PET	An animal PET scanner developed by Dr. Suleman Surti	[40]
Inveon	Siemens Inveon PET	[43]
Metis	Metis PET is commercially provided by Shandong Madic Technology Co.,Ltd	[47]
IRIS	IRIS PET is commercially provided by Medilumine Inc	[46]
ClairvivoPET	ClairvivoPET is a commercial provided by Shimadzu Corp., Japan	[41]
HiPET	A small animal PET scanner developed by Dr. Zheng Gu	[30]
LabPET-12	Scanner developed at University de Sherbrooke, Canada	[42]
ClearPET	ClearPET was manufactured by Raytest Isotopenmessgeraete GmbH, Germany	[44]
Albira Si PET	Bruker Albira Si fully integrated PET/SPECT/CT	[29]
SIAT aPET	A small animal PET developed at Shenzhen Institute of Advanced Technology, China	[31]
β-CUBE	Scanner developed by MOLECUBES, Belgium	[28]
quadHIDAC32	Scanner developed by Oxford Positron Systems	[38]
SimPET-X	A PET insert for simultaneous mouse total-body PET/MR imaging	[13]
Yamaya’s PET	A total-body small animal PET is underdeveloped at Dr. Taiga Yamaya’s Lab, Japan	[21]
Mouse J-PET	A total-body mouse J-PET simulated by Jagiellonian University	[48]
Rat J-PET	A total-body rat J-PET simulated by Jagiellonian University	[48]
HS-BGO PET	A total-body small animal PET with ~ 330-mm axial FOV is underdeveloped at University of California at Davis	[49]
H^2^RS110	A high-resolution (0.5 mm) and high sensitivity PET with 167-mm axial FOV is underdeveloped at University of California at Davis	[12]
H^2^RS160	A high-resolution (0.5 mm) and high sensitivity PET with 254-mm axial FOV simulated at University of California at Davis	[12]
UHS-PET	An over 50% sensitivity PET proposed and simulated by University of California at Davis	[50]

## Abbreviations

ADC: Analog-to-digital converter
ASIC: Application-specific integrated circuit
BGO: Bismuth germanate
CPU: Central processing unit
DAQ: Data acquisition system
DOI: Depth-of-interaction
EW: Energy window
FBK: Fondazione Bruno Kessler
FBP: Filtered back projection
FDG: Fluorodeoxyglucose
FOV: Field-of-view
FPGA: Field-programmable gate array
FWHM: Full width at half maximum
GAGG: Gadolinium aluminum gallium garnet
GPU: Graphics processing unit
GSOZ: Zr-doped gadolinium oxyorthosilicate
GSPS: Giga-samples per second
IDIF: Image-derived input function
LG-SiPM: Linearly graded SiPM
LOR: Line-of-response
LYSO: Lutetium–yttrium oxyorthosilicate
MAPMT: Multi-anode photomultiplier tube
ML: Maximum likelihood
MLEM: Maximum likelihood expectation maximization
MRPC: Multigap resistive plate chamber
MWPC: Multiwire proportional chamber
OSEM: Ordered subset expectation maximization
PCB: Printed circuit board
PET: Positron emission tomography
PMT: Photomultiplier tube
PS-SiPM: Position-sensitive SiPM
PVE: Partial volume effect
RMD: Radiation Monitoring Devices, Inc.
SiPM: Silicon photomultiplier
SNR: Signal-to-noise ratio
SUV: Standard uptake value
TAC: Time-activity curve
TDC: Time-to-digital converter
TOF: Time-of-flight
TSPO: Translocator protein
